# Few differences in psychiatric comorbidities and treatment response among people with anorexia nervosa and atypical anorexia nervosa

**DOI:** 10.1002/eat.24046

**Published:** 2023-09-22

**Authors:** Marley G. Billman Miller, Ayla N. Gioia, Jamal H. Essayli, Lauren N. Forrest

**Affiliations:** 1 Department of Pediatrics, Penn State College of Medicine, Hershey, Pennsylvania, USA; 2 Department of Psychology, Hofstra University, Hempstead, New York, USA; 3 Department of Psychiatry and Behavioral Health, Penn State College of Medicine, Hershey, Pennsylvania, USA

**Keywords:** anorexia nervosa, atypical anorexia nervosa, classification, diagnosis, partial hospitalization program (PHP)

## Abstract

**Objective:**

Little is known about how individuals with atypical anorexia nervosa (AN) respond to eating disorder (ED) treatment in a partial hospitalization program (PHP), nor how certain factors such as trauma, childhood abuse, psychiatric comorbidity, and suicidal thoughts and behaviors might contribute to poor treatment outcomes. Thus, the current study (1) compares prevalence of these factors between individuals with AN and atypical AN upon admission to an ED PHP, (2) compares PHP treatment response between groups, and (3) investigates whether experiencing these factors impacts treatment outcomes.

**Method:**

We conducted a retrospective chart review of young adults admitted to a PHP with AN (*n* = 95) and atypical AN (*n* = 59). Histories of psychiatric comorbidities and adverse childhood experiences were obtained from initial psychiatric evaluations. ED symptoms were assessed at intake and discharge with the Eating Disorder Examination-Questionnaire (EDE-Q).

**Results:**

No significant differences were found at intake in ED symptom severity or prevalence of lifetime trauma, childhood abuse, number of psychiatric diagnoses, or suicidal thoughts and behavior. Symptomatology at discharge also did not differ between groups. Emotional abuse was significantly related to discharge shape and weight overvaluation. No other intake characteristics were significantly related to discharge symptomatology.

**Discussion:**

To our knowledge, this is the first study to compare the prevalence of comorbidities for both AN and atypical AN, as well as differential treatment outcomes for these individuals in a PHP. Results add to growing literature suggesting that, other than weight, AN and atypical AN have few properties that reliably distinguish them from one another.

**Public Significance:**

This study adds to a growing body of literature that raises questions about whether anorexia nervosa (AN) and atypical AN are truly different diagnoses. Our findings suggest these two groups present to treatment in a partial hospitalization program (PHP) with similar ED symptoms, as well as prevalence of lifetime trauma, childhood abuse, suicidal thoughts and behavior, and number of psychiatric comorbidities, and demonstrate similar treatment trajectories in PHP.

## 1 | INTRODUCTION

The *Diagnostic and Statistical Manual of Mental Disorders-5th Edition* (*DSM-5*) categorizes anorexia nervosa (AN) and atypical AN as different eating disorder (ED) types. AN is a fully-specified ED, whereas atypical AN is a subtype of the broader “other specified feeding and eating disorder” category. The diagnostic criteria for AN and atypical AN are highly similar. Both AN and atypical AN are characterized by intense fear of weight gain, body image disturbance, and concerning weight loss (American Psychiatric Association, 2013). Their one notable difference—and the reason they are categorized separately—is body weight. People with AN are underweight compared to national norms, while people with atypical AN may present at any weight other than underweight (Moskowitz & Weiselberg, 2017).

The *DSM*-5 defines AN severity based on a weight-derived metric—body mass index (BMI)—where lower BMI indicates greater illness severity (American Psychiatric Association, 2013). However, despite the fact that adolescents and adults with AN have a lower BMI than those with atypical AN, prior research suggests that both groups experience similar levels of malnutrition, relapse rates, and risk for mortality (Davenport et al., 2015; Garber et al., 2019; Harrop et al., 2021; Moskowitz & Weiselberg, 2017). Further, recent meta-analyses have found that people with AN and atypical AN do not differ on most markers of physical health, eating pathology, or general psychopathology (Abber et al., 2022; Johnson-Munguia et al., 2024), with the exception of greater menstrual disturbance and lower bone mineral density in adolescents and adults with AN relative to atypical AN (Walsh et al., 2022). This growing body of work raises questions about the current classification of AN and atypical AN as distinct disorders, and the utility of low body weight as a criterion that distinguishes these two EDs.

Additional research is warranted to understand whether objectively underweight people with AN actually have a distinct disorder from non-underweight, but severely weight-suppressed, people with atypical AN. Although there are not global differences in eating pathology between individuals with AN and atypical AN, some evidence suggests that adolescents and adults with atypical AN may experience *greater* distress related to eating and body image compared to people with AN (Eiring et al., 2021; Johnson-Munguia et al., 2024; Sawyer et al., 2016). This elevated distress may be due to those with atypical AN not looking “like they have an ED”—i.e., not being emaciated—which may lead family, peers, and healthcare providers to trivialize those with atypical AN’s experiences and ED symptoms (Eiring et al., 2021; Harrop et al., 2023; Sawyer et al., 2016). This, in turn, may lead to fewer adolescents and adults with atypical AN being referred or admitted to higher levels of care for ED treatment (Harrop et al., 2021). Better understanding the similarities and differences between AN and atypical AN has implications for how we conceptualize, label, and diagnose these individuals, which subsequently can impact access to treatment including acceptance into specific treatment programs and insurance coverage (Harrop et al., 2021, 2023; Moskowitz & Weiselberg, 2017).

Despite evidence of increased risk of stigma from peers and providers, there have been few investigations of how individuals with AN and atypical AN might differ in their psychiatric comorbidities and response to treatment (Sawyer et al., 2016; Zanna et al., 2020). Psychiatric comorbidity is not only highly common in EDs (Udo & Grilo, 2019), but is also associated with elevated eating pathology in adults (Lydecker & Grilo, 2021) and higher rates of treatment dropout (Castellini et al., 2018). For example, childhood trauma (Malecki et al., 2018) and abuse (Guillaume et al., 2016) are positively associated with ED pathology in adolescents and adults with AN.

Few studies have empirically compared abuse histories between individuals with AN versus atypical AN (Pauls et al., 2020). Similarly, from a clinical perspective, the presence of suicidal thoughts or behaviors is often seen as indicating more severe psychopathology (Nock et al., 2008), yet we are not aware of any studies comparing lifetime history of suicidal thoughts or behaviors between people with AN versus atypical AN. For studies that have compared comorbidity between people with AN and atypical AN, results have been mixed. Some studies have determined that there are not stark differences between adolescents in these two diagnostic groups (Sawyer et al., 2016), while others have found small differences that call for replication (Pauls et al., 2020; Zanna et al., 2020).

To date, research supports the importance of exploring trauma, childhood abuse, suicidal thoughts, or behaviors, and/or psychiatric comorbidity in predicting treatment outcomes for individuals with AN (Carter et al., 2006; Herzog et al., 1996; Trottier, 2020). Some of these factors have been identified as salient predictors of low body weight, mortality, eating pathology, amenorrhea, and limited psychosocial functioning for both adolescents and adults with AN in higher levels of care, as well as across transdiagnostic ED samples (Herzog et al., 1996; Salbach-Andrae et al., 2009; Vall & Wade, 2015). For example, experiences of suicidal thoughts and behaviors have been associated with worse treatment outcomes for adolescents and adults with AN in inpatient levels of care (Olatunji et al., 2015). Furthermore, childhood abuse has been associated with premature treatment termination in an adult sample of individuals with AN admitted to inpatient care (Carter et al., 2006) and relatedly, lifetime trauma was associated with premature treatment termination in a transdiagnostic sample of adult ED partial hospitalization program (PHP) patients (Trottier, 2020). In some circumstances, however, these factors have driven increased motivation for change in treatment. For example, in one sample of adolescents and adults with EDs attending an ED PHP, comorbid depression moderated treatment outcome (Hayes et al., 2019), such that greater levels of depression were associated with greater *improvement* over the course of treatment. However, despite the role of trauma, abuse, suicidal thoughts, or behaviors, and/or psychiatric comorbidity in predicting both positive and negative treatment outcomes, no studies to our knowledge have investigated whether these clinical characteristics *differentially* impact treatment outcomes for those with AN and atypical AN in higher levels of care. Conducting such an investigation could yield information that may help clinicians better target co-occurring clinical characteristics that influence treatment outcomes.

Accordingly, this study included three aims. Aim 1 compared ED pathology and the prevalence of lifetime trauma, childhood abuse (sexual, physical, and emotional), number of psychiatric comorbidities, and suicidal thoughts and behavior between people with AN and atypical AN upon admission to an ED PHP. Aim 2 compared ED symptom response from PHP admission to discharge between people with AN and atypical AN. Aim 3 identified whether psychiatric comorbidities, childhood abuse, and/or lifetime suicidal thoughts and behaviors interacted with diagnosis to impact response to ED treatment. We hypothesized that, in line with past research, people with AN and atypical AN would show differences in baseline characteristics of psychiatric comorbidities, childhood abuse, and lifetime suicidal thoughts and behaviors, but they would not differ in ED pathology. We also hypothesized that differences in these comorbid factors would impact treatment outcomes, in that individuals who did not experience these adversities would exhibit a greater reduction in ED symptoms throughout treatment.

## 2 | METHOD

### 2.1 | Participants

We conducted a retrospective chart review of patients who were admitted to a PHP for young adults with EDs from October 2017 to September 2021. The chart review start date was chosen given that this was the month and year when ED symptom measures began being systematically administered. The end date was chosen given that this was when analyses were performed for the current study. A total of 95 participants with AN (73.7% restrictive subtype, 26.3% binge/purge subtype) and 59 participants with atypical AN received treatment during this time period. Diagnoses were determined by a licensed psychiatrist during initial evaluation for admission to PHP. All participants exhibited primary symptom presentations of eating restriction and weight suppression. Additionally, 31.2% of the overall sample reported secondary symptoms of binge eating, and 30.7% reported secondary symptoms of purging.^1^ More information on our treatment program can be found in Appendix S1.

Patient age ranged from 16 to 37 years (Table 1).^2^ Most of the sample was White (94%) and identified as female (92.2%). A majority of participants in both groups (95% AN, 88% atypical AN) successfully completed treatment in the PHP and stepped down to outpatient care after discharge (see Appendix S1). This study was approved by the Institutional Review Board of the Penn State College of Medicine (#8749). A full waiver of the informed consent process and authorization for the uses and disclosures of protected health information was obtained, as this research posed no more than minimal harm to subjects.

### 2.2 | Outcome variables

#### 2.2.1 | Eating disorder symptoms and body mass index

Eating disorder symptoms were assessed with the Eating Disorder Examination Questionnaire (EDE-Q; Fairburn, 2008). The EDE-Q is a 28-item self-report questionnaire assessing ED symptoms experienced in the past 28 days. Items are scored on a seven-point rating scale, ranging from 0 (no days) to 6 (every day). The EDE-Q was administered at admission and discharge. Although the EDE-Q was originally specified as having four subscales (Restraint, Eating Concern, Shape Concern, and Weight Concern), this factor structure rarely replicates and there is often strong overlap between subscales (e.g., Shape Concern vs. Weight Concern). An alternative factor structure has been evaluated that includes three subscales: Dietary Restraint, Body Dissatisfaction, and Shape/Weight Overvaluation. This alternative factor structure has been replicated in numerous samples (e.g., Grilo et al., 2015). Thus, at both timepoints, we calculated the EDE-Q Global score and the Dietary Restraint, Body Dissatisfaction, and Shape/Weight Overvaluation subscales to understand both how global and specific ED symptoms changed and/or differed over treatment. The EDE-Q Global score (*α* = .96) and all subscales (*α*s = .88–.91) showed good internal consistency.

BMI was also calculated at admission and discharge using height and weight data, which was obtained by licensed nutritionists as part of the interdisciplinary care within the clinic. Changes in BMI from admission to discharge were inspected only for participants whose treatment team indicated at admission that weight restoration was a treatment goal (AN *n* = 69, atypical AN *n* = 14).

### 2.3 | Predictor variables

Indicators of psychiatric comorbidity were derived from participants’ initial psychiatric evaluations, which they completed with a psychiatrist upon PHP admission. The initial psychiatric evaluation is a semi-structured interview, where all participants are asked about: lifetime traumatic experiences; childhood experiences of physical abuse, emotional abuse, and sexual abuse; and lifetime experiences of suicidal ideation and suicide attempts. Each of these variables was entered into the research database as present or absent.

During the initial psychiatric evaluation, participants may also be diagnosed with a number of comorbid psychiatric disorders, based on the psychiatrist’s assessment. Participants in this sample had comorbid diagnoses of: major depressive disorder, generalized anxiety disorder, obsessive-compulsive disorder, panic disorder, social anxiety disorder, specific phobia, attention-deficit/hyperactivity disorder, bipolar disorder (types 1 and 2), borderline personality disorder, substance use disorder (alcohol and cannabis), insomnia disorder, and posttraumatic stress disorder. Given the lack of standardized assessment of these disorders, we calculated the number of psychiatric disorders (other than their primary ED diagnosis) for each participant and used this as a variable in analyses.

### 2.4 | Data analyses

At admission, missing data ranged from 0% to 8.4%. Of the 154 participants who were admitted, discharge data were available for 111 (72.9%).^3^ Missing data were imputed using multilevel multiple imputation (to account for repeated measurements, *m* = 30) using the jomo R package (Quartagno & Carpenter, 2022). The imputed datasets were read into SPSS, and pooled results are reported for all analyses.

Data were analyzed using IBM SPSS (version 28.0, IBM Corp, 2021). We used Pearson’s chi square analyses and independent-samples *t*-tests to evaluate differences between AN and atypical AN on prevalence of lifetime trauma, childhood abuse (sexual, physical, and emotional), number of psychiatric comorbidities, and suicidal thoughts and behavior at admission.

We used five linear regression models to assess whether participants with AN versus atypical AN differed in treatment response. The outcomes in the models were EDE-Q Global, Dietary Restraint, Body Dissatisfaction, Shape/Weight Overvaluation, and BMI at discharge (note that BMI was an outcome for only the weight restoration group). In all models, diagnosis (AN vs. atypical AN) was the predictor and there were two covariates: (1) admission ED symptoms or BMI (this variable differed across models; in all models, it was the same ED symptom type as the dependent variable, but assessed at admission vs. discharge), which created residual change scores, and (2) length of stay (in weeks) to account for differences in treatment duration. In addition, for any predictor variables that differed by diagnosis (in Aim 2), we planned a priori to include an interaction term between diagnosis and the predictor variable, to understand whether diagnosis interacted with any of the predictor variables to moderate ED symptoms or BMI at discharge. Due to the range of ages represented in this sample, additional models using age as a covariate were also included as Tables S1–S3.

An a priori power analysis was conducted using G*Power 3.1.9.7 (Faul et al., 2007) to determine the minimum sample size required to test hypotheses. Results indicated the required sample size to achieve 80% power for detecting medium effects (Cohen, 1992), at a significance criterion of *α* = .05, was *N* = 88 for chi-square tests, *N* = 126 for independent samples *t*-tests, and *N* = 66 for linear regression. Thus, our sample of *N* = 154 was adequately powered to test study hypotheses.

## 3 | RESULTS

### 3.1 | Between-group comparisons

Table 2 shows the ED symptom scores at admission and discharge by group. At admission, independent samples *t*-tests indicated that people with AN versus atypical AN did not significantly differ on EDE-Q Global scores. Groups also did not differ on admission Dietary Restraint, Shape/Weight Overvaluation, or Body Dissatisfaction subscales. At discharge, participants with atypical AN reported significantly greater body dissatisfaction than those with AN. Groups differed significantly at both admission and discharge on BMI, such that those with atypical AN had a significantly higher BMI at both timepoints than those with AN.

Table 3 shows the baseline comorbidity variables by group. Chi-square tests and *t*-tests showed that participants with AN and atypical AN did not differ on the prevalence of lifetime trauma, childhood abuse (sexual, physical, and emotional), number of psychiatric diagnoses, or presence of suicidal thoughts and behavior at admission.

### 3.2 | Longitudinal results

Given that no baseline comorbidities differed by diagnosis, we specified all regression models to include comorbidities as predictors, and did not include any interactions between comorbidity and diagnosis. Table 4 shows the results of the regression predicting change on the EDE-Q Global. Admission EDE-Q Global scores significantly and positively predicted discharge EDE-Q Global scores. Notably, diagnosis did not significantly predict discharge EDE-Q scores, indicating that people with AN and atypical AN did not show differential EDE-Q Global scores over time. Further, none of the predictor variables nor length of stay significantly predicted discharge EDE-Q Global scores.

Table 5 shows the results of the regressions predicting change on the Dietary Restraint, Shape and Weight Overvaluation, and Body Dissatisfaction EDE-Q subscales. While admission scores significantly and positively predicted discharge scores for all three subscales, diagnosis and length of stay did not significantly predict discharge Dietary Restraint, Shape and Weight Overvaluation, and Body Dissatisfaction. Only one predictor variable, emotional abuse, significantly predicted discharge Shape and Weight Overvaluation, such that those who had not experienced emotional abuse had a greater rate of change (steeper slope) in Shape and Weight Overvaluation than those who had experienced emotional abuse. No other predictor variables significantly predicted Dietary Restraint, Shape and Weight Overvaluation, and Body Dissatisfaction at discharge.

Table 6 shows the results of the regression model predicting change in BMI for those participants with a weight restoration goal. Admission BMI significantly and positively predicted discharge BMI. No other predictor variables significantly predicted BMI at discharge. This means that despite significant differences in BMI at both timepoints, people with AN and atypical AN who had a weight restoration treatment goal experienced similar change in BMI throughout treatment.

## 4 | DISCUSSION

To our knowledge, this is the first study to compare the prevalence of childhood abuse, lifetime suicidal thoughts and behaviors, and number of psychiatric diagnoses between people with AN and atypical AN. Further, we assessed if there were differences in treatment outcomes between individuals with AN and atypical AN in a PHP, and if any comorbidities impacted treatment response. We found that at admission, there were no significant differences on ED symptoms or the prevalence of trauma, physical abuse, emotional abuse, sexual abuse, suicidality (lifetime suicidal ideation, lifetime suicide attempt), or number of psychiatric diagnoses between those with AN versus atypical AN. We also found that the two groups did not differ in the extent to which their ED symptoms changed from PHP admission to discharge. These findings indicate that there are many similarities between participants with AN and atypical AN upon treatment presentation, and that these two groups experience similar PHP treatment outcomes.

People with AN and people with atypical AN reported similar prevalence of childhood abuse, trauma, suicidality, and number of psychiatric diagnoses. The finding of similar prevalence of suicidality and psychiatric comorbidity aligns with some existing literature (Moskowitz & Weiselberg, 2017; Sawyer et al., 2016). However, the finding of similar prevalence of childhood abuse in AN and atypical AN diverged from those of Pauls et al. (2020), which found that adolescents with atypical AN were more likely to experience adverse childhood experiences than those with AN (Pauls et al., 2020). There are at least two potential reasons for this different finding. First, adverse childhood experiences include, but are not limited to, childhood abuse. Thus, it is possible that individuals with AN and atypical AN have similar rates of childhood abuse, while those with atypical AN have higher rates of adverse childhood experiences more broadly than people with AN. Second, Pauls et al. (2020)’s AN sample included only people with AN-restricting subtype, whereas our sample included people with both AN-restricting (73.7%) and AN-binge/purge subtypes (26.3%). People with the AN-restricting subtype typically have lower prevalence of traumatic experiences than people with AN-binge/purge subtype (Longo et al., 2019). Therefore, it is possible that differences in prevalence of adverse childhood experiences between people with AN and atypical AN may depend on which AN subtypes are sampled. Taken together, we found very little evidence that baseline comorbidities differ between patients with AN and atypical AN in a PHP.

Only one variable, emotional abuse, predicted outcomes on the Shape and Weight Overvaluation subscale of the EDE-Q. No other variables predicted outcomes on any EDE-Q subscale, nor BMI for the weight restoration group, at discharge. This is consistent with some previous literature reviews that have found that psychological comorbidities are not consistent predictors of treatment outcomes (Herzog et al., 1996). However, diagnosis (AN vs. atypical AN) and length of stay also did not predict any metric of ED symptoms at discharge. These findings were somewhat surprising, as prior research has found that patients with atypical AN experience significantly greater improvements in treatment than those with AN (Silén et al., 2015; Strober et al., 1999). For instance, Silén et al. (2015) compared treatment outcomes in 47 adolescents with AN or atypical AN and found that adolescents with atypical AN had a shorter duration of treatment (*M* = 1.1 years) than adolescents with AN (*M* = 2.2 years) and were also 4.3 times more likely to achieve recovery than those with AN (Silén et al., 2015). However, outcomes in these studies were defined by weight gain, BMI, resumption of menses, and only one measure assessing attitudes toward ED behaviors (Silén et al., 2015; Strober et al., 1999). When treatment success is determined in part by weight-based metrics, and when weight is the only differentiator of AN versus atypical AN, it makes sense that studies would find faster and/or more favorable treatment response in atypical AN versus AN. To avoid this confound, our study purposely chose to include BMI as an outcome only for individuals with a stated goal of weight restoration; whereas among all participants we assessed cognitive ED symptoms, which are more accurate indicators of ED severity than weight-based measures (Dang et al., 2023).

Overall, our results extend the literature suggesting that, other than weight status, individuals AN and atypical AN have few features that reliably distinguish them from one other. The lack of differences between these two disorders begs the question of whether AN and atypical AN should continue to be diagnosed separately. More broadly, though, questioning whether AN and atypical AN are better conceptualized as a single diagnosis versus discrete diagnoses also brings up the utility of dimensional models of ED psychopathology. Dimensional models of ED psychopathology recognize that discrete ED diagnoses have many similarities with one another, and yet also recognizes that ED presentation of the same diagnosis is heterogeneous across people. Conceptualizing EDs categorically versus dimensionally has specific treatment implications. Categorical conceptualizations often imply that a specific intervention type should be employed for a specific ED diagnosis (e.g., integrative cognitive–affective therapy for bulimia nervosa). Dimensional conceptualizations imply identifying individualized target areas (e.g., food avoidance, regular eating, purging, weight restoration) and employing evidence-based intervention components for an individual’s specific target areas (e.g., exposure for food avoidance in conjunction with emotion regulation skills for purging; Forbush et al., 2017; Forbush et al., 2018). Preliminary evidence suggests that a specific dimensional model, the Hierarchical Taxonomy of Internalizing Dimensions for EDs, is more predictive of change in ED symptoms compared to DSM-5 categorial diagnoses (Forbush et al., 2018). Whether dimensional conceptualizations improve treatment outcomes and/or reduce disparities in receiving ED treatment for those with atypical AN (Harrop, 2020; Veillette et al., 2018) are important directions for future research.

This study has several limitations. Our sample was primarily White, female, and from the central Pennsylvania region, which limits the generalizability of findings. Also, our sample is limited to a PHP setting, and results may not generalize to other types of treatment. Follow-up data were not collected after PHP discharge, so we are limited to studying change throughout participants’ several weeks in PHP. While some studies have examined outcomes in other types of treatment settings for those with AN (e.g., Madden et al., 2015), we do not know the long-term outcomes of receiving this treatment for those with atypical AN, nor do we know how long-term outcomes may differ in other settings. Future studies should systematically compare long-term treatment outcomes of these groups within multiple treatment settings. Comorbid factors evaluated in this study were not all encompassing (e.g., we could not consider differences based in prevalence of specific disorders or disorder types) and were collected through a non-standardized clinical interview. Duration of illness was not systematically collected in this sample, limiting our ability to control for this variable. Also, due to sample-size limitations, we were underpowered to detect small effects, which is important to consider when making conclusions about finding no differences between the AN and atypical AN group. Multiple statistical tests were utilized, and future replication studies with larger samples should correct for multiple comparisons. Further, replication research with a larger, more diverse sample and use of standardized instruments to assess psychiatric comorbidity is warranted to better understand the prevalence of psychiatric comorbidity and its impact on treatment outcomes in people with AN and atypical AN.

Additionally, given that individuals with atypical AN are less likely to be referred to higher levels of care (Harrop et al., 2021), we are unable to determine how many individuals with atypical AN were *not* referred to our treatment facility. It is possible that less severe or psychiatrically complex individuals with atypical AN are less likely to be identified as having an ED, and that the subset of participants with atypical AN in our treatment facility do not represent the broader group of people with atypical AN. Lastly, changes in weight were evaluated for individuals who had a stated goal of weight restoration, but the amount of weight gain prescribed by their treatment team was not systematically recorded in the clinical or research database. Therefore, we were unable to assess for changes in BMI relative to weight suppression and treatment goal weights.

In conclusion, we found that participants with AN and atypical AN had similar psychiatric comorbidity and treatment outcomes in an ED PHP. This work adds to a growing body of literature that raises questions about whether AN and atypical AN are truly two separate diagnoses. Future research should continue to explore dimensional approaches to conceptualizing individuals with AN and atypical AN, which may be more predictive of treatment outcomes than the current categorical model.

## Supplementary Material

Supplementary Material

Supplementary Tables

## Figures and Tables

**TABLE 1:** Demographics.

Variable	AN, *n* = 95 % (*n*)	Atypical AN, *n* = 59 % (*n*)	*χ* ^2^	*p*
Gender				
Man	6.3 (6)	5 (8.5 (5))	.863	.649
Woman	92.6 (88)	54 (91.5 (54))		
Trans man	1.1 (1)	0 (.0 (0))		
Race/ethnicity				
White	90.5 (86)	91.5 (54)	2.57	.766
Black/African American	1.1 (1)	.0 (0)		
Hispanic/Latino	1.1 (1)	3.4 (2)		
Asian	3.2 (3)	1.7 (1)		
Other	1.1 (1)	.0 (0)		
Unavailable	3.2 (3)	3.4 (2)		
Goal of weight restoration (admission)	72.6 (69)	23.7 (14)	35.03	<.001
Reported binge eating (admission)	26.1 (23)	40.0 (20)	2.86	.091
Reporting purging (admission)	30.2 (26)	31.4 (16)	.20	.889
	*M (SD)*	*M (SD)*	*t*	*p*
Age in years	20.55 (3.33)	20.19 (3.78)	.62	.537
Length of stay in weeks	7.68 (4.80)	6.45 (2.88)	1.98	.050

*Note*: Prevalence of binge eating at admission was determined if EDE-Q #14 ≥ 1. Prevalence of purging at baseline was determined using EDE-Q #16 or #17 ≥ 1.

* *p* < .05

** *p* < .01

*** *p* < .001.

**TABLE 2:** Mean scores and standard deviations of the EDE-Q Global and subscales at admission and discharge.

	Admission					Discharge				
Outcome	AN	Atypical AN	*t*	*p*	*d*	AN	Atypical AN	*t*	*p*	*d*
EDE-Q Global	2.94 (1.60)	3.37 (1.68)	‒1.52	.131	‒.260	2.16 (1.50)	2.56 (1.37)	‒1.44	.152	‒.278
EDE-Q Dietary Restraint	3.27 (2.17)	3.92 (2.16)	‒1.75	.082	‒.304	1.87 (1.99)	1.78 (1.61)	.26	.794	.052
EDE-Q Shape/Weight Overvaluation	4.14 (1.83)	4.48 (1.86)	‒1.08	.284	‒.187	2.90 (2.00)	3.66 (1.94)	‒1.96	.053	‒.386
EDE-Q Body Dissatisfaction	4.15 (1.93)	4.63 (1.66)	‒1.50	.136	‒.261	2.81 (2.11)	3.75 (1.94)	‒2.35	.021	‒.462
BMI	18.71 (1.94)	23.05 (4.12)	‒7.46	<.001	‒1.47	19.70 (1.83)	23.39 (3.66)	‒6.98	<.001	‒1.39

*Note*: Independent samples *t*-tests were used to examine statistical differences at admission and discharge between groups.

Abbreviations: BMI, body mass index; *d*, Cohen’s *d* effect size; EDE-Q, Eating Disorder Examination Questionnaire.

**TABLE 3:** Percentage of sample endorsing adverse childhood experiences, suicidality, and psychiatric comorbidity.

Variable	AN, *n* = 95 % (*n*)	Atypical AN, *n* = 59 *n* (%)	χ2	*p*
Physical abuse	16.8 (16)	12.1 (7)	.64	.423
Emotional abuse	12.6 (12)	22.4 (13)	.40	.529
Sexual abuse	26.3 (25)	31.0 (18)	2.52	.112
Trauma	22.1 (21)	29.3 (17)	1.00	.317
Lifetime suicide ideation	33.7 (32)	39.0 (23)	.45	.505
Lifetime suicide attempt	13.7 (13)	20.3 (12)	1.19	.276
	*M (SD)*	*M (SD)*	*t*	*p*
# Psychiatric diagnoses	1.55 (1.18)	1.69 (1.21)	‒.75	.456

*Note*: Pearson’s chi square analyses and independent-samples *t*-tests to statistically evaluate differences between groups.

**TABLE 4:** Results of linear regression model assessing whether diagnosis (AN vs. atypical AN) and/or psychiatric comorbidity predicts change in global eating disorder symptoms.

Outcome	*b*	SE	*t*	*p*	*sr^2^*
EDE-Q Global at admission	.58	.07	8.44	<.001	.612
Length of stay (weeks)	.01	.03	.39	.697	.027
Diagnosis (reference = AN)	–.06	.21	–.28	.781	–.018
Physical abuse	–.31	.26	–.88	.380	–.059
Emotional abuse	.29	.33	1.14	.254	.074
Sexual abuse	.01	.34	.03	.978	.002
Trauma	–.19	.28	–.67	.500	–.045
Suicidal ideation	.29	.27	1.07	.286	.075
Suicide attempt	.19	.34	.57	.568	.037
# Psychiatric diagnoses	.15	.09	1.63	.104	.111

*Note*: Diagnosis: AN was coded as 0, atypical AN was coded as 1. All predictor variables: absent coded as 0, present coded as 1.

Abbreviations: *b*, unstandardized beta; *SE*, standard error; *sr^2^*, squared semi-partial correlation.

**TABLE 5:** Results of linear regression models assessing whether diagnosis (AN vs. atypical AN) and/or psychiatric comorbidity predicts change in EDE-Q empirical subscales.

	Dietary Restraint					Shape/Weight Overvaluation					Body Dissatisfaction				
Outcome	*b*	*SE*	*t*	*P*	*sr^2^*	*b*	*SE*	*t*	*P*	*sr* ^***2***^	*b*	*SE*	*t*	*P*	*sr^2^*
EDE-Q subscale at admission	.35	.08	4.62	<.001	.403	.60	.08	8.04	<.001	.565	.58	.08	7.22	<.001	.509
Length of stay (weeks)	.03	.04	.87	.387	.071	.01	.03	.36	.718	.025	.06	.03	1.70	.089	.117
Diagnosis (reference = AN)	-.31	.32	-.96	.340	-.080	.20	.27	.76	.446	.051	.27	.29	.95	.343	.066
Physical abuse	-.27	.57	-.47	.638	-.042	-.93	.52	-1.79	.075	-.138	-.95	.56	1.68	.095	-.136
Emotional abuse	.52	.41	1.28	.203	.106	.72	.36	2.01	.045	.141	.44	.37	1.19	.234	.083
Sexual abuse	-.20	.50	-.39	.695	-.032	.10	.44	.22	.823	.015	.24	.47	.52	.606	.036
Trauma	-.36	.42	-.85	.398	-.070	-.40	.36	-1.13	.258	-.076	-.41	.37	-1.13	.260	-.075
Suicidal ideation	-.03	.41	-.07	.948	-.005	.59	.35	1.66	.097	.118	.34	.39	.88	.381	.065
Suicide attempt	.18	.53	.33	.740	.027	-.03	.46	-.06	.951	-.004	.34	.49	.69	.493	.048
# Psychiatric diagnoses	.24	.14	1.75	.081	.145	.20	.12	1.65	.099	.114	.21	.13	1.61	.108	.114

*Note*: Diagnosis: AN was coded as 0, atypical AN was coded as 1. All predictor variables: absent coded as 0, present coded as 1.

Abbreviations: *b*, unstandardized beta, *SE*, standard error, *sr*^2^, squared semi-partial correlation.

**TABLE 6:** Results of linear regression model assessing whether diagnosis (AN vs. atypical AN) and/or psychiatric comorbidity predicts change in BMI for those with a goal of weight restoration at admission.

Outcome	*b*	*SE*	*t*	*p*	*sr* ^2^
BMI at admission	.83	.08	10.70	<.001	.653
Length of stay (weeks)	.02	.03	.94	.347	.054
Diagnosis (reference = AN)	.44	.37	1.21	.227	.073
Physical abuse	.03	.45	.06	.953	.004
Emotional abuse	–.34	.33	–1.02	.307	–.063
Sexual abuse	.59	.47	1.25	.213	.077
Trauma	–.02	.36	–.06	.950	–.004
Suicidal ideation	.15	.34	.43	.668	–.025
Suicide attempt	–.63	.49	–1.28	.200	–.078
# Psychiatric diagnoses	–.06	.12	–.48	.631	–.028

*Note*: Diagnosis: AN was coded as 0, atypical AN was coded as 1. All predictor variables: absent coded as 0, present coded as 1.

Abbreviations: *b*, unstandardized beta, *SE*, standard error, *sr*^2^, squared semi-partial correlation.

## Data Availability

The data that support the findings of this study are available on request from the corresponding author. The data are not publicly available due to privacy or ethical restrictions.

## Footnotes

1 Binge-eating and purging behaviors at admission were derived from self-reported responses on the EDE-Q. Binge eating was defined as ≥1 time on EDE-Q #14, and purging was defined as ≥1 times on EDE-Q #16 or #17.

2 Although the PHP is referred to as a “young adult” program, the program admits patients as young as 16, given that the other treatment program available to these patients is for children.

3 We examined the proportion of people with AN and atypical AN who did vs. did not complete discharge assessments. For AN, 31.6% (*n* = 30) did not complete discharge assessments. For atypical AN, 22.0% did not complete discharge assessments (*n* = 13).
