# Supplementary material for: Few differences in psychiatric comorbidities and treatment response among people with anorexia nervosa and atypical anorexia nervosa
Source: Int J Eat Disord. Author manuscript; Available in PMC 2025 Feb 24. (PMC11849127; doi:10.1002/eat.24046)
Supplement: Supplementary Material [file NIHMS2041280-supplement-Supplementary_Material.docx]

**Supporting Information**

**Impact of Predictor Variables on Treatment Dropout**

Pearson’s Chi-square tests were used to statistically compare reason for stepdown from PHP between groups. No significant difference (p=.137) between groups was found in reason for stepdown, with a majority (95% AN, 88% AAN) successfully completing the PHP and stepping down to outpatient care.

Pearson’s Chi-square tests were also used to determine if those who experienced childhood abuse, trauma, and suicidal thoughts and behaviors experienced greater levels of dropout, and a one-way ANOVA was used to determine if those who dropped out of treatment possessed a greater number of psychiatric diagnoses. Since there were no significant differences between groups on these variables, the entire sample was evaluated, and between-group comparisons were not utilized. No significant differences in the reason for dropout were found by history of childhood emotional abuse (*p*=.527), sexual abuse (*p*=.062), trauma (*p*=.846), suicidal ideation (*p*=.858), suicide attempt (*p*=.094), or number of psychiatric diagnoses at baseline (*p*=.345). There was a significant difference in reason for dropout by history of physical abuse, such that a greater proportion of those who had a history of physical abuse dropped out of program (26.1%, *p*<.001) compared to those who did not have a history of physical abuse (3.8%).

**Treatment Program**

  The partial hospitalization program (PHP) for young adults with eating disorders at Penn State Health provides treatment for an average of six hours per day, five days per week. The PHP is conducted by an interdisciplinary team of dietitians, therapists, pediatricians, nurse-practitioners, and psychiatrists. The PHP includes multiple group and individual therapy sessions, and family therapy as warranted. Patients receive two supervised meals and one snack per day. The PHP adheres to a flexible, cognitive-behavioral model. Cognitive-behavioral interventions utilized in the PHP include: meal planning, self-monitoring, psychoeducation, exposure therapy, cognitive restructuring, and distress tolerance skills. The PHP treats young adults with anorexia nervosa, bulimia nervosa, avoidant/restrictive food intake disorder, binge-eating disorder, and other specified feeding or eating disorders. Additional descriptions about the program’s approach to treatment can be found in prior publications (Essayli et al., 2021; Ortiz et al., 2023).
