# Supplementary material for: Few differences in psychiatric comorbidities and treatment response among people with anorexia nervosa and atypical anorexia nervosa
Source: Int J Eat Disord. Author manuscript; Available in PMC 2025 Feb 24. (PMC11849127; doi:10.1002/eat.24046)
Supplement: Supplementary Tables [file NIHMS2041280-supplement-Supplementary_Tables.docx]

| Supplementary Table 1. *Results of linear regression model assessing whether diagnosis (AN vs. atypical AN) and/or psychiatric comorbidity predicts change in global eating disorder symptoms with age as a covariate* | | | | | |
| --- | --- | --- | --- | --- | --- |
| Outcome | *b* | SE | *t* | *p* | *sr^2^* |
| EDE-Q Global at admission | 0.58 | 0.07 | 8.40 | <.001 | .607 |
| Length of stay (weeks) | 0.01 | 0.03 | 0.39 | .698 | .027 |
| Age (years) | -0.00 | 0.03 | -0.04 | .971 | -.002 |
| Diagnosis (Reference = AN) | -0.06 | 0.21 | -0.28 | .780 | -.019 |
| Physical abuse | -0.31 | 0.35 | -0.88 | .382 | -.059 |
| Emotional abuse | 0.29 | 0.26 | 1.14 | .254 | .074 |
| Sexual abuse | 0.01 | 0.33 | 0.03 | .980 | .002 |
| Trauma | -0.19 | 0.28 | -0.67 | .506 | -.045 |
| Suicidal ideation | 0.29 | 0.27 | 1.07 | .286 | .075 |
| Suicide attempt | 0.19 | 0.34 | 0.58 | .565 | .037 |
| # Psychiatric diagnoses | 0.15 | 0.10 | 1.60 | .110 | .110 |
| *Note*. Diagnosis: AN was coded as 0, atypical AN was coded as 1. All predictor variables: Absent coded as 0, Present coded as 1. *b* = unstandardized beta, *SE* = standard error,  *sr^2^* = squared semi-partial correlation | | | | | |

| Supplementary Table 2. *Results of linear regression models assessing whether diagnosis (AN vs. atypical AN) and/or psychiatric comorbidity predicts change in EDE-Q empirical subscales with age as a covariate* | | | | | | | | | | | | | | | |
| --- | --- | --- | --- | --- | --- | --- | --- | --- | --- | --- | --- | --- | --- | --- | --- |
|  | *Dietary Restraint* | | | | | *Shape/Weight Overvaluation* | | | | | *Body Dissatisfaction* | | | | |
| Outcome | *b* | SE | *t* | *p* | *sr^2^* | *b* | SE | *t* | *p* | *sr^2^* | *b* | SE | *t* | *p* | *sr^2^* |
| EDE-Q subscale at admission | 0.35 | 0.08 | 4.62 | <.001 | .401 | 0.60 | 0.08 | 8.09 | <.001 | .567 | 0.58 | 0.08 | 7.22 | <.001 | .508 |
| Length of stay (weeks) | 0.03 | 0.04 | 0.86 | .389 | .071 | 0.01 | 0.03 | 0.23 | .819 | .016 | 0.05 | 0.03 | 1.60 | .111 | .111 |
| Age (years) | -0.00 | 0.05 | -0.05 | .960 | -.005 | 0.05 | 0.05 | 1.13 | .260 | .090 | 0.04 | 0.04 | 0.79 | .429 | .060 |
| Diagnosis (reference = AN) | -0.31 | 0.32 | -0.95 | .342 | -.080 | 0.22 | 0.27 | 0.84 | .401 | .056 | 0.29 | 0.29 | 1.00 | .320 | .069 |
| Physical abuse | -0.27 | 0.57 | -0.47 | .641 | -.041 | -0.98 | 0.51 | -1.91 | .058 | -.144 | -0.98 | 0.56 | -1.75 | .081 | -.140 |
| Emotional abuse | 0.52 | 0.41 | 1.27 | .203 | .106 | 0.73 | 0.36 | 2.06 | .040 | .143 | 0.45 | 0.37 | 1.22 | .223 | .085 |
| Sexual abuse | -0.20 | 0.50 | -0.40 | .693 | -.032 | 0.13 | 0.44 | 0.29 | .775 | .019 | 0.26 | 0.47 | 0.56 | .577 | .039 |
| Trauma | -0.36 | 0.42 | -0.84 | .402 | -.070 | -0.45 | 0.36 | -1.28 | .202 | -.085 | -0.45 | 0.37 | -1.22 | .224 | -.081 |
| Suicidal ideation | -0.03 | 0.41 | -0.06 | .949 | -.005 | 0.58 | 0.35 | 1.63 | .103 | .116 | 0.33 | 0.39 | 0.86 | .389 | .063 |
| Suicide attempt | 0.18 | 0.53 | 0.34 | .735 | .028 | -0.10 | 0.46 | -0.22 | .825 | -.015 | 0.29 | 0.49 | 0.58 | .562 | .041 |
| # Psychiatric diagnoses | 0.24 | 0.14 | 1.73 | .084 | .145 | 0.18 | 0.12 | 1.50 | .135 | .104 | 0.20 | 0.13 | 1.50 | .135 | .107 |
| *Note*. Diagnosis: AN was coded as 0, atypical AN was coded as 1. All predictor variables: Absent coded as 0, Present coded as 1. *b* = unstandardized beta, *SE* = standard error,  *sr^2^* = squared semi-partial correlation | | | | | | | | | | | | | | | |

| Supplementary Table 3. *Results of linear regression model assessing whether diagnosis (AN vs. atypical AN) and/or psychiatric comorbidity predicts change in BMI with age as a covariate for those with a goal of weight restoration at admission* | | | | | |
| --- | --- | --- | --- | --- | --- |
| Outcome | *b* | SE | *t* | *p* | *sr^2^* |
| BMI at admission | 0.83 | 0.08 | 10.67 | <.001 | .652 |
| Length of stay (weeks) | 0.03 | 0.03 | 0.98 | .330 | .057 |
| Age (years) | -0.01 | 0.04 | -0.32 | .752 | -.019 |
| Diagnosis (Reference = AN) | 0.43 | 0.37 | 1.15 | .252 | .069 |
| Physical abuse | 0.02 | 0.45 | 0.05 | .958 | .003 |
| Emotional abuse | -0.31 | 0.34 | -0.93 | .352 | -.058 |
| Sexual abuse | 0.56 | 0.49 | 1.14 | .256 | .071 |
| Trauma | -0.00 | 0.38 | -0.01 | .996 | -.001 |
| Suicidal ideation | 0.14 | 0.34 | 0.40 | .691 | -.023 |
| Suicide attempt | -0.60 | 0.51 | -1.16 | .246 | -.072 |
| # Psychiatric diagnoses | -0.05 | 0.13 | -0.40 | .688 | -.023 |
| *Note*. Diagnosis: AN was coded as 0, atypical AN was coded as 1. All predictor variables: Absent coded as 0, Present coded as 1.  *b* = unstandardized beta, *SE* = standard error,  *sr^2^* = squared semi-partial correlation | | | | | |
